# Pamphlets Received

**Published:** 1881-03

**Authors:** 


Pamphlets received: Consumption and Tuberculosis: Notes on their Treatment by the Hypophosphites. By J. A. McArthur, M.D., Fellow of the Massachusetts Medical Society.

Anæmia in Infancy and Early Childhood. By A. Jacobi, M.D., Clinical Professor of Diseases of Children in the College of Physicians and Surgeons, New York.

Hemiopia: Mechanism of its Causation on the Theory of Total Decausation of the Optic Nerve Fibres in the Optic Tract at the Chiasma. By Wm. Dickinson, M.D., New York.

The Development of the Osseous Callus in Fractures of the Bones of Man and Animals. By Henry D. Marcy, A M., M D., member of American Medical Association, etc. Reprint from Transactions American Medical Association for 1880.

The Relations of Goitre to Pregnancy and Derangements of the Generative Organs of Women. By Edward W. Jenks, M.D., L.L D. Reprint from the *American Journal of Obstetrics* and Diseases of Women and Children. Vol. XIV. No. 1, January, 1880.

Remarks on Syphilis. By Walter Coles, M.D. Reprint from Transactions of St. Louis Medical Society, in St. Louis *Medical and Surgical Journal*, January 1881.

Annual Announcement of the Courses of Lectures Delivered in the Philadelphia School of Anatomy. 1881.

Morton's Pocket Series Aphorisms in Fractures. By R D. Cowling, A.M., M.D., Professor of Principles and Practices of Surgery in the University of Louisville, Kentucky.

Annual Announcement of the Medical College of the Pacific. 1881.

